# An overview of Frontiers in Research Metrics and Analytics

**DOI:** 10.3389/frma.2024.1420385

**Published:** 2024-05-16

**Authors:** Chaomei Chen, Zaida Chinchilla-Rodríguez, Yi Zhang, Ben Daniel, Yuya Kajikawa, Dietmar Wolfram

**Affiliations:** ^1^Drexel University, Philadelphia, PA, United States; ^2^Institute for Public Goods and Policies (IPP), Spanish National Research Council (CSIC), Madrid, Spain; ^3^University of Technology Sydney, Sydney, NSW, Australia; ^4^University of Otago, Dunedin, New Zealand; ^5^Tokyo Institute of Technology, Meguro, Japan; ^6^University of Wisconsin–Milwaukee, Milwaukee, WI, United States

**Keywords:** meta-knowledge, research landscape, co-cited journals, *Scientometrics*, research metrics and analytics

## Introduction

Frontiers in Research Metrics and Analytics (FRMA) was launched in 2016 as an open-access journal to provide a forum for learning, evaluating, and improving research and scholarship in a wide range of disciplines. FRMA aims to provide a stimulating and inspirational forum not only for scholars devoted to advancing theories and instruments of how research should be done and how it can be done efficiently but also for researchers to apply and integrate available conceptual frameworks and enabling technologies in their own practice of research. The overarching mission of FRMA embraces insightful and profound contributions from researchers who are specialized in different disciplines to accomplish the same goal—reaching research excellence at a new level. Just as astronomers study stars and galaxies through increasingly powerful telescopes, researchers can study the dynamics of their own field of research through quantitative and qualitative methods. The more we learn about how research has been done and can be done better, the more researchers and stakeholders will benefit from increased efficiency and effectiveness of research. Advancing such meta-knowledge of research has profound and far-reaching implications in our society.

FRMA currently consists of 6 sections of specialty, including integral parts of research such as assessment, evaluation, scholarly communication, and strategic management of research and innovation. We asked our specialty editors to select exemplary works published in the past few years in their sections so that we can share with a broader community and foster broader and profound interactions. Our editors are particularly attracted by a collection of 7 intriguing FRMA publications. We hope these highlighted publications will illustrate the types of insightful and inspirational contributions for researchers across multiple disciplines.

## A broader context

FRMA has published 369 articles so far, including original research, review, perspective, methods, and technical report. To better understand the overarching insights across individual contributions from a diverse range of perspectives and specialties, we will first explore the intellectual neighborhood of FRMA in a broader context of journals that have been frequently cited by FRMA authors and then at a more granular level how FRMA publications are distributed among research articles that have been cited in similar contexts.

### Cohorts of co-cited journals

To take into account the potential scholarly impact of published articles, we focus on FRMA publications that have at least one citation and they must cite some published articles. There are at least two bibliographic databases covering FRMA publications, namely, Digital Science's Dimensions and Scopus. With our inclusion criteria, we retrieved 358 FRMA publication records from Dimensions. These 358 FRMA articles form the core dataset, which will be subsequently expanded to an expanded dataset. Articles in the core dataset collectively cited 8,717 unique references. The core articles as a whole have been cited by 1,157 publications.

**Figure 1:**
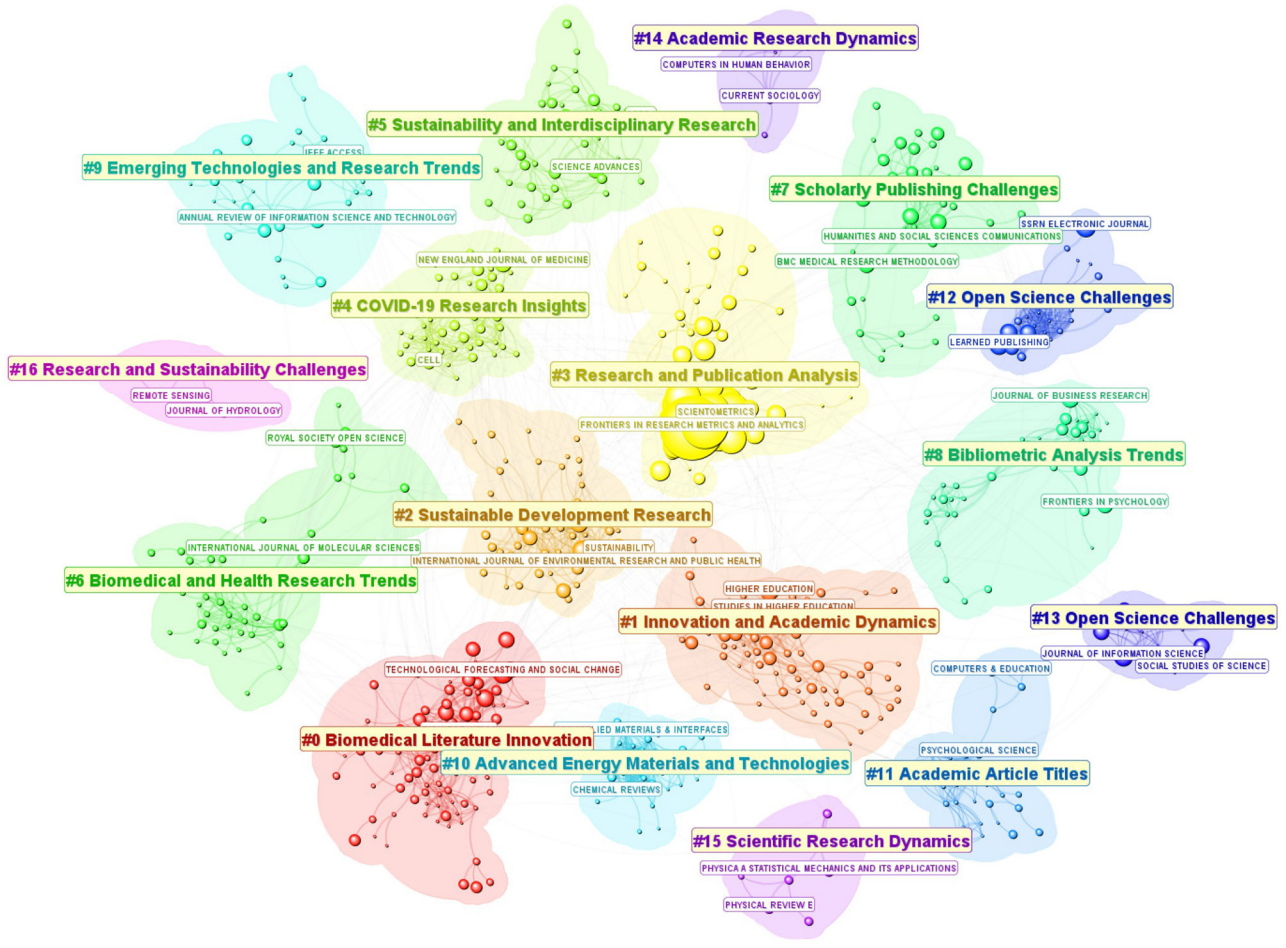
A network visualization of journals cited by the FRMA articles. Journal titles of the two most prevalent journals in each cluster are shown. FRMA itself appears in Cluster #3 Research and Publication Analysis.

Figure 1 shows a network visualization of journals cited by the FRMA articles. Journals are grouped into distinct clusters based on how often they are cited together. Each cluster is labeled based on the most common themes of citing articles, which are all FRMA articles. Several recurring themes such as research, innovation, interdisciplinary research, research trends, and challenges clearly reflect FRMA's overall scope and specific focuses of individual sections. FRMA itself belongs to Cluster #3 Research and Publication Analysis—this is the context FRMA has been cited, which is inline with the FRMA's mission.

There are journals that are frequently cited along with FRMA as they appear in the vicinity of FRMA and their citations have increased rapidly since 2020 when FRMA experienced a significant expansion (see Supplementary Figure S1). Among the 6 journals, *Scientometrics, Journal of the Association for Information Science and Technology* (JASIST), and *Journal of Informetrics*, are highly cited, indicating that they share some common interests with FRMA.

### FRMA publications in context

**Figure 2:**
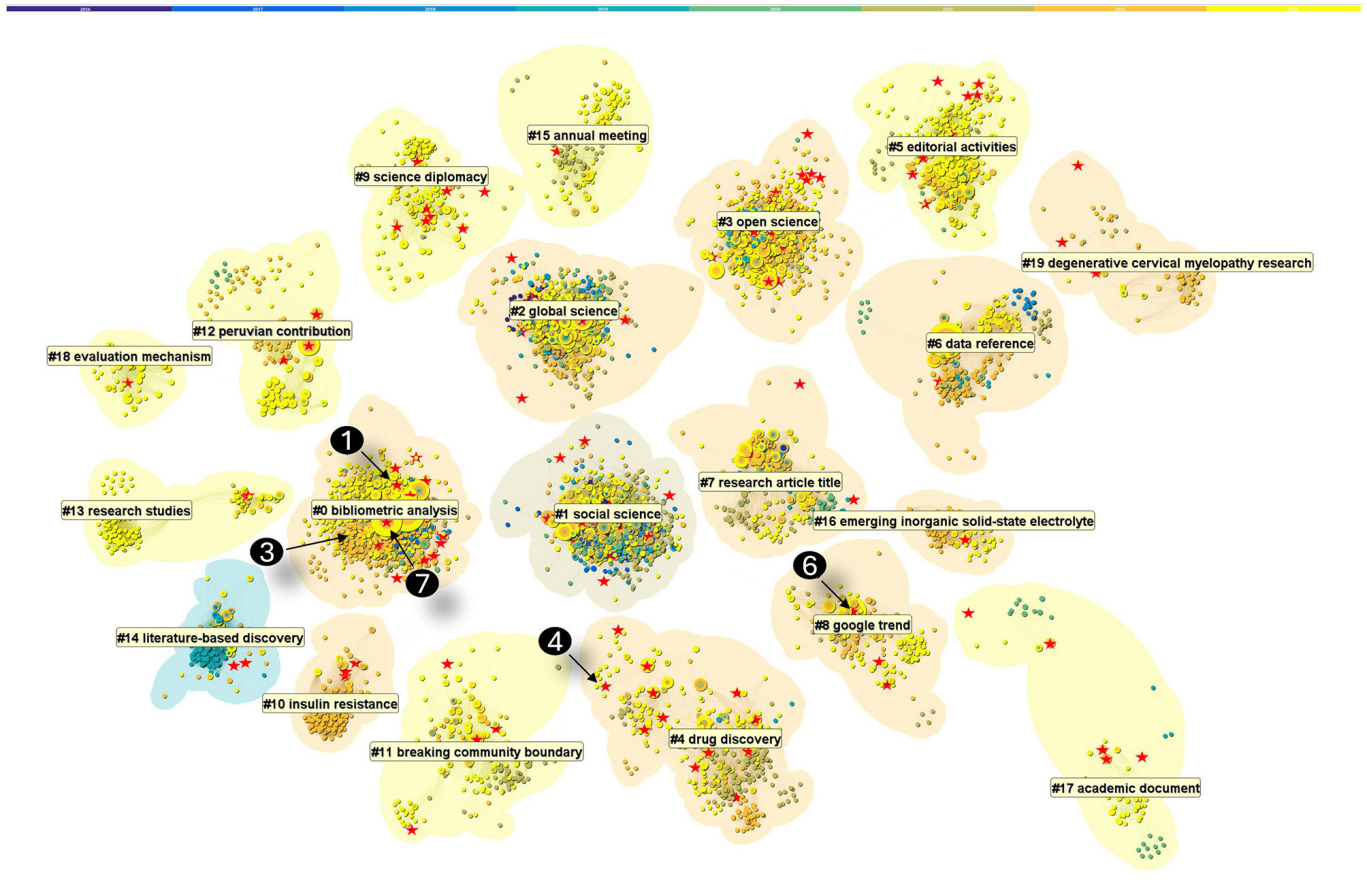
An overview of major clusters of references cited by the expanded dataset, featuring 6,059 references cited between 2016 and 2023. Red stars are FRMA publications. Numbered stars are FRMA exemplars.

To reveal how FRMA publications are distributed among other publications cited in similar thematic areas, we constructed an expanded dataset with 2,425 publications (2016–2023) that cited the references cited by FRMA publications. The expanded dataset covers contributions from 7,913 authors across 994 journals. They collectively cited 99,849 references. We visualized a network of co-cited references based on the citing behavior of top 1,000 most cited publications per year during the period of 2016 and 2023. In addition, each reference must be cited at least twice each year to be eligible. The resultant network featured 6,076 references and 189,988 co-citation links (see Figure 2).

Each node is depicted with its citation tree ring, which is color coded from inside out. One can get some general idea of a cluster from its color patterns. For example, clusters near the center with a wider range of colors spanning multiple years have a relatively longer history than clusters further out with more homogeneous colors of more recent years. The footprint of FRMA in this broader context is shown by the distribution of red stars. Each red star represents a FRMA publication. For example, FRMA publications have a strong presence in #0 bibliometric analysis, #1 social science, and #3 open science, whereas a relatively weaker presence in clusters such as #6, #10, and #11, primarily COVID-19 related topics.

References marked by Chen (2020), Cabezas-Clavijo and Torres-Salinas (2021), Rovetta (2021), Porter and Hook (2022), Puljak et al. (2022), Kalenzi (2022), and Perez-Molina and Loizides (2023) are the exemplar works identified by our editors. They appeared in three different clusters, indicating their distinct roles and impacts on subsequent research.

One way to gauge the scholarly impact of a research publication is how fast it attracts attention in terms of citation burst. The strength of a citation burst is how steep its citations increase. The duration of a citation burst is how long its citations sustain a fast increase. Supplementary Figure S2 highlights 6 FRMA publications among 66 references with the strongest citation bursts that last 4 years or longer. The 6 FRMA articles were published between 2016 and 2018, prior to the rapid growth of the journal since 2020 and leading to the exemplar works that would appear soon after.

In summary, the mission of FRMA is to provide a stimulating and open platform for the advancement of meta-knowledge of research and innovation as well as new ways to carry out high-quality research that have broad impacts assessment, evaluation, communication, and strategic management. The exemplary works highlighted by our specialty editors provide examples of how researchers from different areas of expertise may contribute to the shared mission.

## Author contributions

CC: Conceptualization, Investigation, Visualization, Writing—original draft, Writing—review & editing. ZC-R: Conceptualization, Investigation, Writing—original draft, Writing—review & editing. YZ: Conceptualization, Investigation, Writing—original draft, Writing—review & editing. BD: Conceptualization, Investigation, Writing—original draft, Writing—review & editing. YK: Conceptualization, Investigation, Writing—original draft, Writing—review & editing. DW: Conceptualization, Investigation, Writing—original draft, Writing—review & editing.
