# Supplementary figures and images for: An overview of Frontiers in Research Metrics and Analytics
Source: Front Res Metr Anal. 2024 May 16;9:1420385. doi: 10.3389/frma.2024.1420385 (PMC11146195; doi:10.3389/frma.2024.1420385)

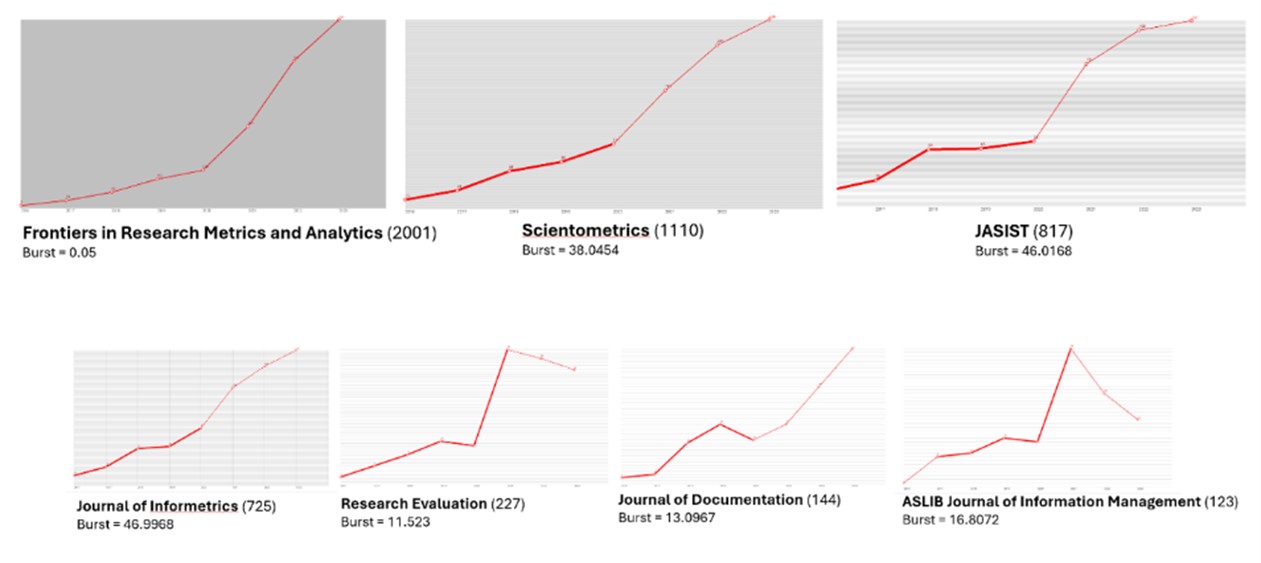

Supplement: Supplementary file 1 [file Image_1.JPEG]

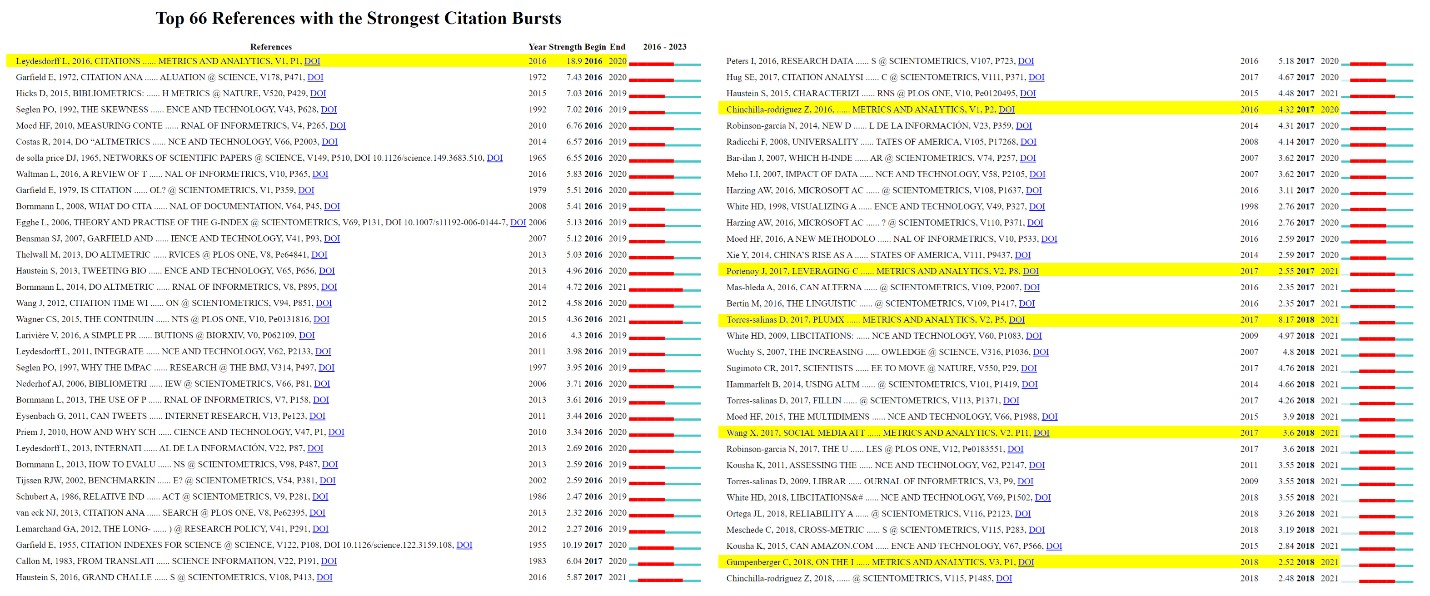

Supplement: Supplementary file 2 [file Image_2.JPEG]
